# Optimization Design of Key Mold Components for Slab Quality Improvement: Clamping Mechanism and Narrow Copper Plate

**DOI:** 10.3390/ma19050862

**Published:** 2026-02-25

**Authors:** Wenxue Wang, Yu Wang, Mingjun Qiu, Bo Yang, Xiaoping Liang, Xinqiang Li, Chenggong Yao, Zhengchun Li, Jun Huang

**Affiliations:** 1 College of Materials Science and Engineering, Chongqing University, Chongqing 400044, China; 2 China National Heavy Machinery Research Institute Co., Ltd., Xi’an 710032, China; 3 School of Energy and Environment, Inner Mongolia University of Science and Technology, Baotou 014010, China

**Keywords:** continuous casting, mold, clamping mechanism, cooper plate, optimization design

## Abstract

**Highlights:**

**What are the main findings?**

- Integrated mold optimization proposed. Disc spring-hydraulic clamp ensures robust casting.
- Narrow-face copper plate outer chamfer designed based on solidification principles.
- External chamfer reduces corner defects, extends life 20%, improves yield 0.1%.

**What are the implications of the main findings?**

- Extends narrow-face life by 20%, reducing maintenance and replacement costs.
- Reduces corner defects and improves yield, enhancing product quality and competitiveness.
- Provides a technical approach for future caster mold design and engineering practice.

**Abstract:**

The surface quality and production efficiency of continuous-casting steel slabs are predominantly determined by the performance of the mold. To address slab corner defects and enhance operational stability, this study systematically optimized two key components: the broad-face clamping mechanism and the narrow-face copper plate. A disk spring–hydraulic composite clamping mechanism was designed and subjected to mechanical analysis to ensure sufficient and reliable clamping force under high-load casting conditions. Meanwhile, based on the principle of solidification shrinkage, an external chamfer structure for the narrow-face copper plate was proposed to improve heat transfer uniformity at the slab corner. Engineering design calculations and practical application in an export-oriented wide-and-heavy slab continuous-casting project (specification: 250 mm × 2500 mm) demonstrated that the optimized clamping mechanism provides enhanced structural rigidity, while the new narrow-face copper plate effectively mitigates corner cracks and reduces wear. This integrated design approach significantly improves slab surface quality and extends component service life, yielding substantial economic benefits.

## 1. Introduction

The continuous-casting mold, as the core stage for the initial solidification of molten steel, directly determines the surface quality and internal structure of the cast slab, making it a critical piece of equipment that affects both the production efficiency of the continuous caster and the final product quality [1]. With the growing demand for high-grade steels in downstream industries—particularly the increased casting proportion of crack-sensitive steel grades such as peritectic and near-peritectic steels—controlling slab corner cracks (e.g., longitudinal face cracks, star cracks) has become a prominent challenge restricting product quality and smooth operation [2]. Research indicates that the formation of such defects results from the complex coupling between thermal stress concentration during the metallurgical process and the mechanical condition of the mold, rooted fundamentally in the uniformity of heat transfer within the mold cavity and the mechanical stability of the assembly between copper plates [3,4].

Currently, wide-slab continuous casting widely employs split-type molds, whose inner cavity is formed by assembling two broad-face copper plates and two narrow-face copper plates, constituting a typical four-wall assembly structure [5]. Such molds must fulfill two core functions: firstly, during high-temperature, high-casting-speed operations, the clamping mechanism must provide constant and sufficient compressive force to the copper plates to maintain extremely narrow joint gaps, preventing molten steel leakage and ensuring overall rigidity [6,7]; secondly, to accommodate different cross-sectional specifications, their narrow-face copper plates must allow for rapid and accurate online width adjustment and replacement [8]. Consequently, the design performance of the clamping mechanism is fundamental to guaranteeing the mold’s function as a “rigid container,” and its reliability directly relates to the risk of breakout and the quality of the slab oscillation marks [7,9].

In terms of heat transfer, conventional narrow-face copper plates typically feature a right-angle design, which leads to a sharp increase in cooling intensity at the slab corner region, forming a two-dimensional heat transfer corner zone [10]. In this zone, the solidifying shell grows too rapidly, resulting in highly concentrated thermal stress that readily induces transverse and longitudinal corner cracks [11]. To mitigate this issue, For the former, a variety of strategies have been developed and widely implemented. These include adjusting mold cooling water flow and temperature distribution, optimizing casting speed and superheat, modifying mold oscillation parameters (frequency, amplitude, and non-sinusoidal characteristics), and tailoring the physicochemical properties of mold fluxes (viscosity, melting temperature, and basicity) [12,13]. Such methods, which focus on regulating heat transfer and lubrication behavior without altering the mold structure, have proven effective in reducing crack incidence and are often preferred due to their flexibility, rapid implementation, and lower cost. However, their control capability is inherently constrained by the fixed geometric and thermal design of the mold cavity; under the increasingly stringent casting conditions required for high-grade crack-sensitive steels, these measures alone often fail to fully eliminate corner defects and may compromise productivity or slab surface quality when pushed to their limits [14,15]. Among these, the internally chamfered (or beveled) narrow-face copper plate, by altering the corner geometry, optimizes heat flux distribution to some extent and has been proven effective in reducing corner cracks [15]. However, this solution also has notable limitations: the mechanical strength of the chamfered region is reduced, making it more susceptible to wear and thermal deformation under high-temperature friction. This leads to shortened service life of the copper plate and increased maintenance costs, thereby constraining its economic benefits.

In summary, relying solely on process adjustments or isolated modifications to the copper plate profile makes it difficult to fundamentally reconcile the contradiction between mechanical constraint and heat flow control. Therefore, from the perspective of overall mold system design, seeking coordinated optimization of mechanical structure and cooling/heat transfer represents an inevitable direction for overcoming current technical bottlenecks [4,8]. Based on solidification shrinkage theory and mechanical simulation analysis, this study proposes an integrated systematic solution: First, a new clamping mechanism based on a disk spring–hydraulic composite principle is designed, aiming to achieve adaptive maintenance of clamping force and high-rigidity locking under high-load conditions, thereby providing a stable mechanical environment for the copper plates. Second, an innovatively designed narrow-face copper plate with an external chamfer is proposed. Rather than directly machining the working face of the copper plate, this design alters its back-profile to create a progressively varying air gap between the copper plate and the cooling water jacket in the corner region. This air gap, serving as an adjustable thermal resistance, enables “soft regulation” of the cooling intensity at the slab corner, promoting uniform longitudinal distribution of heat flow along the corner. Consequently, the goal of crack suppression is achieved without excessively compromising the structural integrity of the copper plate. Through design optimization, the service life of the narrow-face copper plate is extended by nearly 20%, and the metallic yield is increased by approximately 0.1%.

This paper details the conceptual origins, principles of mechanics and heat transfer, specific structural implementation, and the industrial application results of this coordinated optimization design on a 250 mm × 2500 mm wide-and-heavy slab continuous caster. This study aims to provide a novel mold system design solution for the high-quality continuous casting of high crack-sensitive steel grades, one characterized by high reliability, long service life, and excellent slab quality performance.

## 2. Systematic Optimization Methodology and Component Design

This study employs a systematic design methodology that integrates mechanical analysis, heat transfer principles, and engineering validation to enable collaborative optimization of the following two closely coupled subsystems: the clamping mechanism, which ensures mechanical structural stability, and the narrow-face copper plates for regulating solidification heat transfer behavior.

### 2.1. Collaborative Design and Mechanical Performance Optimization of Hybrid Disk Spring–Hydraulic Clamping Mechanism

#### 2.1.1. Structure and Operating Principle

**Figure 1:**
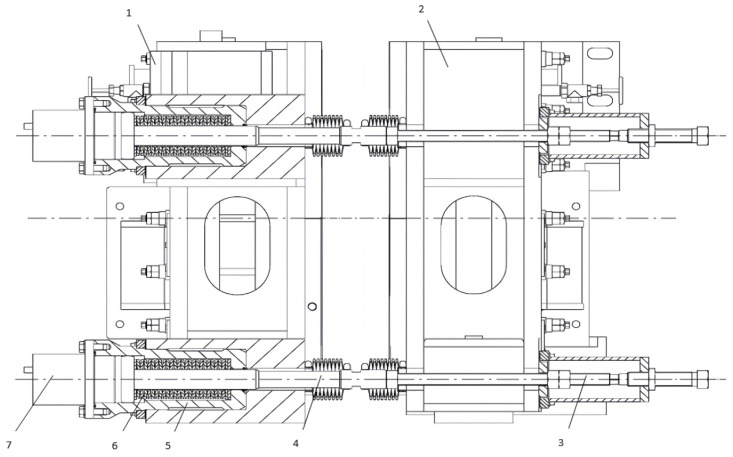
Structure and installation method of the clamping mechanism: 1. outer arc side cooling water tank; 2. inner arc side cooling water tank; 3. adjusting device; 4. pull rod; 5. sleeve; 6. disk spring; 7. hydraulic cylinder.

As illustrated in Figure 1, the mold clamping mechanism designed in this study adopts a hybrid operational principle of “clamping via disk springs, releasing via hydraulic pressure.” It primarily consists of core components including the adjustment device, tie rods, sleeves, disk spring stacks, and hydraulic cylinders. It should be noted that Figure 1 shows a schematic of the unilateral mechanism. A complete mold system requires two identical sets of this mechanism to be symmetrically arranged, acting on both sides of the mold respectively, to ensure balanced clamping force and structural symmetry.

During clamping mode, the disk springs (6) are pre-compressed, exerting a continuous tensile force on the pull rods (4). This force is transmitted through the sleeves (5) to draw the outer and inner arc cooling water tanks (1, 2) toward the broad-face copper plates, thereby maintaining a tight assembly and minimizing the narrow-face joint gap. The direction of the clamping force and component movement is inward (toward the mold cavity), as indicated by solid arrows. During width adjustment or replacement, the hydraulic cylinders (7) act counter to the disk springs. When pressurized, they push the pull rods (4) outward, further compressing the disk springs and releasing the clamping force. This outward movement (shown by dashed arrows) disengages the narrow-face assembly, allowing for rapid and precise repositioning. Regarding heat flow, cooling water enters the cooling water tanks (1, 2) and flows through channels on the back surface of the copper plates, removing heat conducted from the solidifying shell. For the externally chamfered narrow-face copper plate, a progressively varying air gap is intentionally formed between the copper plate back and the cooling water jacket at the corner region. This gap acts as an adjustable thermal resistance, moderating the local heat flux and achieving “soft cooling” at the slab corner (indicated by graded heat flow arrows).

The core design concept of this mechanism lies in utilizing the preload of the disk springs to achieve reliable mechanical locking. Its operational workflow is as follows: the disk spring stack is pre-installed inside the hydraulic cylinder barrel and is guided and positioned by the tie rod. By tightening the rear lock nut, the piston is driven to compress the disk spring stack, thereby generating a clamping force that pushes the inner arc side (moving side) copper plate towards the outer arc side (fixed side) copper plate. This ultimately realizes the stable pressing of the two narrow-face copper plates by the two wide-face copper plates, forming a rigid mold cavity. When the springs are compressed to the predetermined load (i.e., preload), they are secured by the locking element, and the mechanism enters a stable clamping state. Conversely, the releasing process is driven by the hydraulic system: hydraulic oil is fed into the cylinder barrel, pushing the piston to generate a hydraulic force opposite to the direction of the disk spring preload, thereby overcoming the spring force and forcibly opening the moving side to provide the necessary operational space for online width adjustment or replacement of the narrow-face copper plates.

#### 2.1.2. Establishment of the Mechanical Model and Calculation of Key Parameters

**Figure 2:**
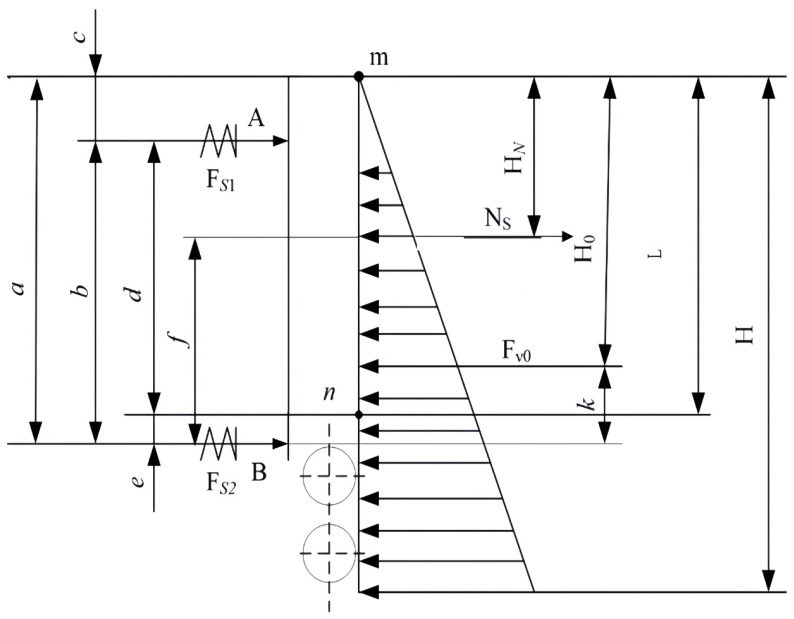
Stress diagram of the mold broad-face copper plates (inner and outer arc sides).

To ensure the reliability of the clamping mechanism under conditions of high casting speed and high molten steel static pressure, a force model for the wide-face copper plate under casting conditions was established. Two assumptions are made here: first, the mold is completely filled with molten steel; second, the force from the water tank acting on the sliding carriage can be considered as an additional friction force [16]. Based on this approach, taking one side of the copper plate as the analysis target, its stress schematic is shown in Figure 2 [1]. Key geometric parameters include the height of the resultant force action point for the static pressure of the molten steel (*H*), the action point position of the spring force from the clamping mechanism, and the distances between it and the support points (*a*, *b*, *c*, *f*, *g*, *j*, *k*).

Each letter in the above schematic is represented as follows: *H* is the height of the hydrostatic steel pressure within mold (including foot rolls); *H*_0_ is the height of the resultant force point of hydrostatic steel pressure; *F_s_*_1_ is the clamping force of the upper disk spring; *F_s_*_2_ is the clamping force of the bottom disk spring; *F_vo_* is the resultant force of the hydrostatic steel pressure; *N_s_* is the friction of the cooling water tank on the slide carriage; *m* is the upper supporting point of the wide and narrow sides of the copper plate; *n* is the bottom supporting point of the wide and narrow sides of the copper plate; *a* is the distance from the top surface of the copper plate to the action point of the bottom disk spring; *b* is the distance of the action point between the upper and bottom disk springs; *c* is the distance from the top surface of the copper plate to the action point of the upper disk spring; *f* is the distance from the action point of the bottom disk spring to the slide carriage; *g* is the distance from the action point of the upper disk spring to the slide carriage; *j* is the distance from the action point of the upper disk spring to the resultant point of the hydrostatic steel pressure; *k* is the distance from the action point of the bottom disk spring to the resultant point of the hydrostatic steel pressure; and *A* is the position of the action point of the upper disk spring.

Taking the 250 mm × 2000 mm cross-section specification targeted in this study as an example, the structural parameters shown in Figure 2 are defined as listed in Table 1.

The static pressure of molten steel during casting *F_V_*_0_ can be calculated using the following formula:

(1) $$F_{V0}=\frac{\gamma \cdot H^{2}\cdot B}{2}$$

where γ is the specific gravity of molten steel, here γ = 7 g/cm^3^.

Therefore, the static pressure of molten steel at positions A and B in Figure 2, denoted as *P_A_*_0_ and *P_B_*_0_, is given by the following equations:

(2) $$P_{A0}=\frac{F_{V0}}{2}\cdot \frac{k}{b}$$

(3) $$P_{B0}=\frac{F_{V0}}{2}-P_{A0}$$

The frictional force of the cooling water tank on the sliding carriage *N_S_* is calculated using the following equation:

(4) $$N_{S}=P_{a}\cdot \mu_{c}$$

where *P_a_* is the force caused by the self-weight of the water tank (considering cooling water), here estimated as 4800 kg, and *μ_c_* is the friction coefficient, here 0.2.

The cooling water tank is supported by sliding brackets at both ends; thus, the frictional force on one side is *N_S_*/2. The forces at points A and B are expressed as follows:

(5) $$P_{AN}=\frac{N_{S}}{2}\cdot \frac{f}{b}$$

(6) $$P_{BN}=\frac{N_{S}}{2}-P_{AN}$$

Considering the preload from the disk springs and summarizing the two aforementioned components, the preload forces at points A and B are calculated as follows:

(7) $$\sum P_{A}=P_{A0}+P_{AN}$$

(8) $$\sum P_{B}=P_{B0}+P_{BN}$$

Based on the principle of moment equilibrium, taking moments about the support point, the minimum clamping force required to balance the operational loads can be derived. Calculations indicate that due to the trapezoidal distribution of the molten steel static pressure along the height, the load acting on the lower part of the mold is significantly greater than that on the upper part. Accordingly, the required disk spring preloads for the upper and lower sections of the mechanism, *F_S_*_1_ and *F_S_*_2_, can be determined by Equations (9) and (10), respectively:

(9) $$F_{s1}=K_{s}\cdot \sum P_{A}$$

(10) $$F_{S2}=K_{s}\cdot \sum P_{B}$$

where *K_S_* is the design safety factor, set to 1.5.

Based on the aforementioned formulas, the calculated stress parameters are listed in Table 2. The calculation results indicate that *F_S_*_2_ is significantly greater than *F_S_*_1_, with a ratio between them of approximately 2.2:1. This mechanical characteristic directly informed the subsequent adoption of a differentiated configuration strategy for the disk spring stacks, described as “looser at the top, tighter at the bottom,” to achieve structural stress optimization and efficient material utilization.

#### 2.1.3. Design and Performance Optimization of Key Components

Based on the aforementioned mechanical analysis, targeted design and optimized selection were conducted for each core component. The selection of disk springs followed the national standard GB/T 1972-2005 [17], and considering constraints such as installation space and bolt strength, the A series (model A100-1) disk spring was ultimately chosen. Its basic parameters are listed in Table 3.

**Figure 3:**
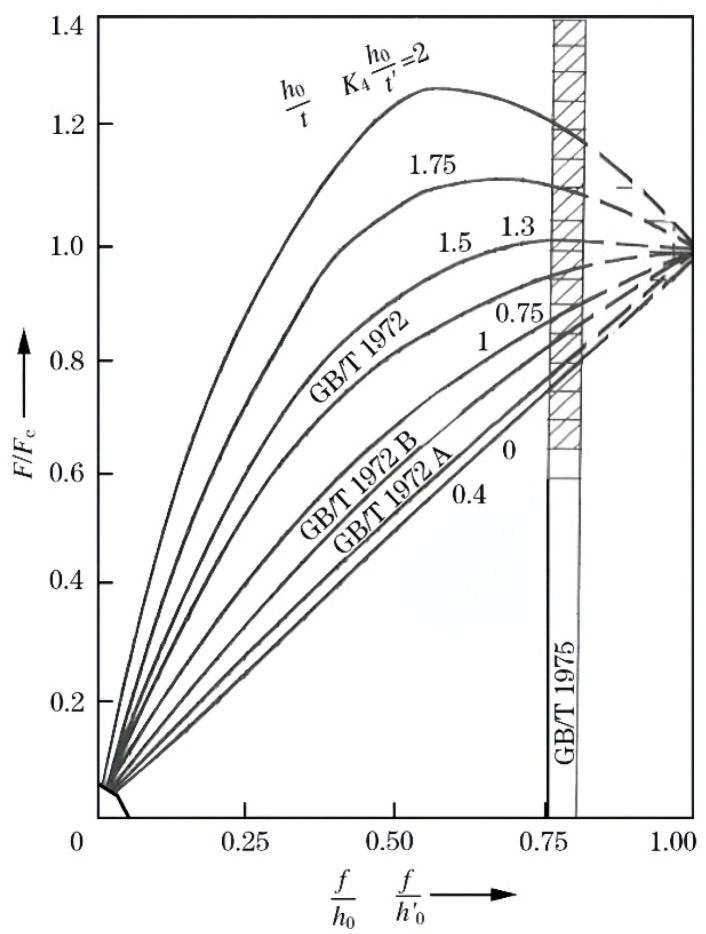
Disk spring performance curve [17].

When the deflection of this spring model satisfies *f* ≤ 0.75 *h*_0_ (where *h*_0_ is the maximum allowable deflection), the relationship between load and deformation is approximately linear, as shown in Figure 3, demonstrating stable performance.

To meet the different load requirements of the upper and lower sections, distinct combination forms are adopted: the upper assembly, which bears a smaller load, employs a “stacked in parallel” combination of 10 disk springs. This combination increases the total deflection to 10 times that of a single spring while maintaining the load-bearing capacity of a single spring, thereby reducing sensitivity to manufacturing and assembly tolerances. The preload force provided by the springs is as shown in Equation (11).

(11) $$F_{S11}=\frac{f^{\prime }}{0.75h_{0}}\cdot 4800$$

The upper spring assembly is set with a pre-compression deformation of 7.5 mm (0.75 mm per individual disk), resulting in a calculated preload force of 21,818.2 N, which meets the design specifications while maintaining an appropriate safety margin. The lower component, which bears the primary load, utilizes a compound arrangement of 20 disk springs, incorporating both series and parallel stacking configurations. This combination effectively increases both the load-bearing capacity and the total allowable deformation. With a pre-compression deformation set at 8.0 mm, the preload force provided by the lower spring assembly, calculated via Equation (11), is 46,545.5 N. This value slightly exceeds the theoretical requirement *F_S_*_2_, thereby ensuring sufficient clamping reliability.

**Figure 4:**
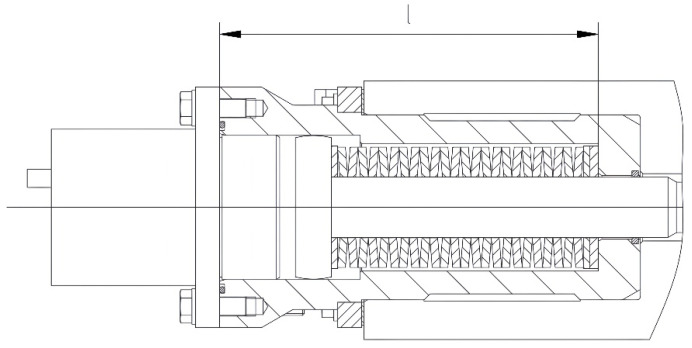
Hydraulic cylinder barrel structure for the clamping mechanism.

The optimal design of the hydraulic cylinder barrel and its stroke is determined as follows: the barrel length *L*_1_ is jointly defined by the preload height of the disk spring set, its free height, and the hydraulic stroke, as illustrated in Figure 4. The design of the hydraulic stroke must balance two key factors: firstly, it must provide sufficient mold opening (typically 5–8 mm) to facilitate operation and maintenance; secondly, the additional compression imposed on the disk springs must be confined within their optimized working range to prevent premature spring failure. In this design, the hydraulic stroke (i.e., the additional spring compression) is strictly confined to within 0.35 *h*_0_ [18]. This ensures that the disk springs remain within the favorable linear elastic region even after unloading, thereby guaranteeing their fatigue life and long-term performance stability.

The disk spring–hydraulic composite clamping mechanism designed in this section employs a differentiated “loose top, tight bottom” disk spring configuration to scientifically balance the non-uniformly distributed load along the mold height direction, achieving a unification of structural lightweight design and high rigidity. The optimized hydraulic stroke ensures operational convenience while preserving the core performance and service life of the disk springs. This design has been successfully applied in an export-oriented continuous-casting machine project for 250 mm × 2500 mm wide thick slab production. Industrial practice demonstrates that the mechanism can provide sustained, stable, and sufficient clamping force under high-load casting conditions, effectively suppressing the tendency for copper plate seam opening. This lays a solid mechanical foundation for uniform oscillation marks on the slab surface and the prevention of breakout risks.

### 2.2. Design and Optimization of Outer Chamfer Structure for Narrow-Face Copper Plate

#### 2.2.1. Analysis of the Causes of Slab Corner Defects and Wear Mechanism of the Copper Plate

**Figure 5:**
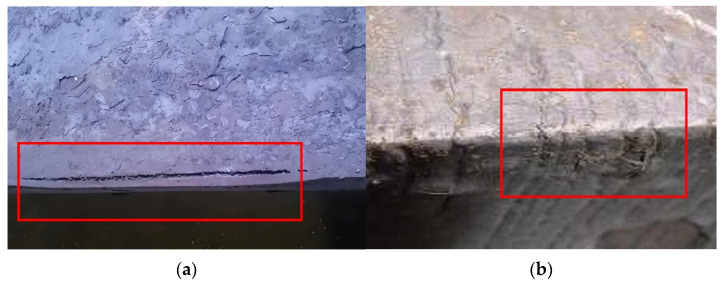
Slab corner defects (actual size): (**a**) longitudinal corner crack; (**b**) depression and transverse corner crack.

In continuous casting production, particularly during the casting of crack-sensitive steel grades, slab corner defects such as longitudinal/transverse corner cracks, star cracks, and depressions may occur, as illustrated in Figure 5. These defects represent critical issues affecting both product quality and production stability. The root cause lies in the concentration of thermal stress resulting from intense two-dimensional heat transfer in the corner region [10]. When the carbon content of the molten steel falls within the peritectic reaction range (0.09–0.15%), the significant volume contraction associated with the δ → γ phase transformation, combined with excessively rapid cooling and uneven solidification shell growth at the corner, leads to highly concentrated contraction stresses that readily initiate cracks [19]. Additionally, the right-angle design of traditional narrow-face copper plates exacerbates uneven cooling at the corner. Process- and equipment-related factors, such as improper primary/secondary cooling configuration, nozzle biased flow, and inadequate mold taper, further contribute to non-uniform solidification shell thickness, amplifying local stress levels. Severe cooling and friction also accelerate wear at the corner of the copper plate, increasing the risks of copper penetration and breakout incidents.

Industrial autopsy of used narrow-face copper plates reveals that wear is predominantly concentrated at the corner region, with the maximum depth occurring approximately 30–50 mm below the meniscus. This localized wear pattern is attributed to the thermomechanical behavior of the solidifying shell. Under conventional right-angle design, the corner region experiences intensified two-dimensional heat transfer, leading to a prematurely thickened and low-temperature shell with higher strength and hardness. This harder shell exerts more aggressive abrasive and adhesive wear on the opposing copper surface during mold oscillation and slab withdrawal. In contrast, the externally chamfered design moderates corner cooling via the backside air gap, resulting in thinner, more uniform shell growth and reduced contact pressure at the corner. Consequently, frictional wear is substantially alleviated, contributing to the observed 20% extension in narrow-face copper plate service life.

Therefore, while optimizing process parameters can alleviate these defects to a certain extent, a fundamental solution requires innovative design and optimization of the structure of the narrow-face copper plate—the core component determining the initial heat transfer conditions.

#### 2.2.2. Design Concept and Thermodynamic Principles of the Outer Chamfer Structure

**Figure 6:**
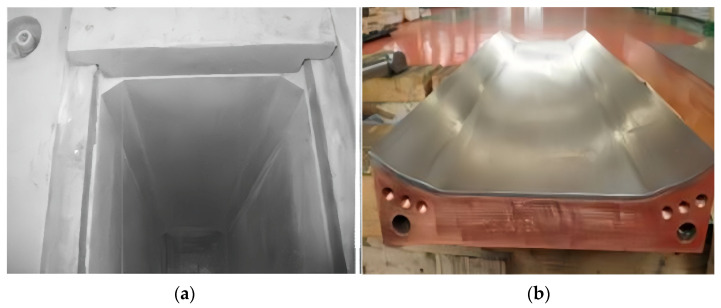
Internal chamfer of the copper mold: (**a**) inner chamfer on mold copper; (**b**) inner chamfer copper plate.

To overcome the limitations of traditional right-angle designs and internal chamfer solutions (such as reduced mechanical strength and accelerated wear), this study abandons the approach of directly machining the working face. Instead, it innovatively proposes an outer chamfer structure design for the narrow-face copper plate, as shown in Figure 6. The core concept is to create a controllable air gap between the slab corner and the copper plate by modifying the geometry of the copper plate backside. This air gap increases linearly along the casting direction (longitudinally) and serves as a “buffer zone” for regulating thermal resistance, thereby achieving “soft” and “gradient” control over the cooling intensity at the corner.

As shown in Figure 1, the narrow-face copper plate is positioned at the center of the mold assembly, flanked by the inner and outer arc cooling water tanks (1, 2) and connected to the clamping mechanism via pull rods (4), sleeves (5), and disk springs (6). The proposed externally chamfered structure is located at the corner region of this narrow-face copper plate, with its geometric configuration depicted through a locally enlarged detail or the progressively varying back-gap in the schematic.

Mechanically, the narrow-face copper plate experiences continuous clamping force from the disk springs (6) during operation. This force is transmitted through the pull rods (4) and sleeves (5) to the cooling water tanks and ultimately to the back face of the narrow-face copper plate, ensuring tight contact with the broad-face plates. Although the external chamfer does not alter the overall load path, the local material removal or back-gap design slightly reduces the stiffness at the corner. Under thermal expansion, this promotes a more compliant contact state that helps alleviate stress concentration.

Thermally, the core innovation lies not in modifying the working face geometry but in engineering the backside contact condition. As illustrated in Figure 6, an inclined outer chamfer is machined on the lower corner region of the copper plate backside, causing the contact area between the copper plate and the cooling water jacket to gradually decrease downward along the casting direction. This produces a continuously varying air gap. Owing to the much lower thermal conductivity of air relative to copper, this gap introduces an adjustable thermal resistance that actively moderates the local cooling intensity. Below the meniscus, the thermal resistance increases nearly linearly, delaying and smoothing heat extraction at the corner. This “corner soft cooling” effectively counteracts the localized overcooling typically induced by two-dimensional heat transfer, allowing the solidification shell growth rate at the corner to better synchronize with that at the wide-face center. As a result, thermal stress caused by uneven contraction is substantially reduced [14,20]. Importantly, this heat transfer optimization is achieved without compromising the integrity or mechanical strength of the copper plate working face, thereby avoiding the accelerated wear and thermal deformation associated with conventional face-chamfering approaches. Consequently, both slab corner quality is improved and the service life of the narrow-face copper plate is significantly extended.

#### 2.2.3. Determination and Optimization of Key Structural Parameters

**Figure 7:**
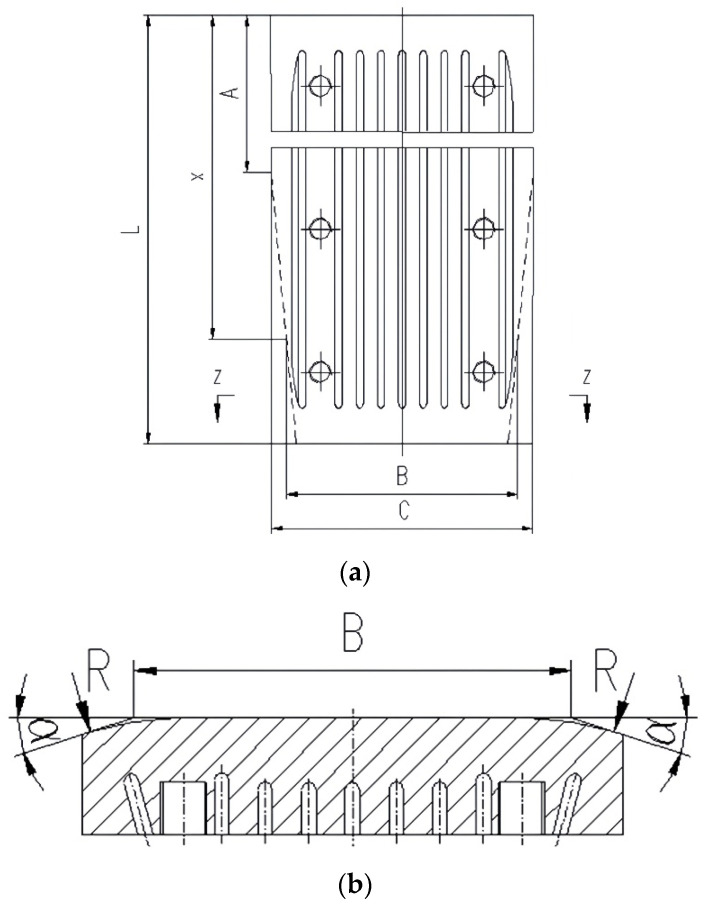
Outer chamfer on narrow copper mold: (**a**) front view of outer chamfer copper plate; (**b**) Z-Z section view of outer chamfer copper plate.

The design of the outer chamfer structure hinges on determining three core geometric parameters: the chamfer starting height *A*, the chamfer width *B*, and the chamfer angle *α*. These parameters require calculation and optimization based on the solidification shrinkage characteristics of the specific steel grade. Their determination relies on a regression analysis of the solidification shrinkage model for the target steel grade within the mold, as illustrated in Figure 7.

The shrinkage allowance is calculated based on the solidification shrinkage characteristics of molten steel within the mold. According to the shrinkage equation, outer chamfers with an angle *α* are machined on both side corners of the narrow copper plate along the casting direction. The chamfering starts from the upper edge of the copper plate over a distance *A* and is also applied across the width *B* on both sides of the narrow copper plate. In this configuration, the gap between the slab corner and the mold corner increases linearly downward, and the thermal resistance rises accordingly. This achieves uniform and gradual cooling of the slab corner, thereby minimizing or eliminating slab corner defects and ensuring slab quality.

Based on slab solidification calculations, outer chamfer machining is performed on both side corners of the lower section of the mold’s narrow-face copper plate. The machining parameters *A* and *B*, as well as the angle *α*, are calculated using Equations (12)–(14):

(12) $$A=\frac{a}{k^{2}}+b$$

(13) $$B=C-2k\sqrt{\frac{\left(x-A\right)}{v}}$$

(14) $$\alpha =\frac{1}{2}\tan^{-1}\cdot \frac{k}{150}$$

where the parameters in above equations are represented as follows: *A* is the distance from the upper edge of the copper plate to the machined chamfer, mm; *x* is the dimension of the upper edge of the copper plate, mm; *B* is the plane width after chamfering in height *x*, mm; *α* is the chamfering angle, °; *a* is the linear coefficient, mm/min; *b* is the linear constant, mm; *k* is the steel solidification coefficient, mm^2^/min; *v* is the design normal casting speed, mm/min; and *C* is the lower exit width of the narrow copper plate, mm.

Furthermore, to ensure structural reliability and avoid stress concentration, the intersection line between the chamfered surface and the working face of the copper plate must be machined into a transition fillet [21]. Based on empirical knowledge and finite element stress analysis, the fillet radius *R* should be greater than or equal to 50 mm. A larger *R* value effectively distributes the stress on the copper plate under high temperature and frictional loads, preventing crack initiation from sharp corners and ensuring the structural integrity of the copper plate during long-term service [22].

The outer chamfer design for the narrow-face copper plate proposed in this section fundamentally addresses heat transfer path regulation. Through ingenious geometric modification of its backside, it achieves optimized cooling at the slab corner, providing an innovative equipment solution that synergistically resolves the conflicting issues of corner cracking and copper plate wear.

## 3. Comprehensive Engineering Verification and Analysis of the Coordinated Optimization Solution

This chapter aims to conduct comprehensive industrial validation and performance evaluation of the proposed coordinated optimization design scheme for the mold. By integrating the disk spring–hydraulic composite clamping mechanism and the outer-chamfered narrow-face copper plate into an actual continuous casting production line, their performance in improving slab quality, enhancing equipment reliability, and generating comprehensive economic benefits is systematically investigated. Furthermore, their operational mechanisms are explored from the perspective of mechanical and thermal coupling.

### 3.1. Industrial Application Performance of the Disk Spring–Hydraulic Composite Clamping Mechanism

The disk spring–hydraulic composite clamping mechanism, designed based on the “loose top, tight bottom” differentiated configuration concept, has been implemented in the industrial application of an export-oriented 250 mm × 2500 mm wide thick slab continuous caster project. By precisely matching the non-uniform load distribution along the mold height direction, this mechanism significantly enhances overall rigidity while achieving structural lightweighting. Industrial practice confirms that this optimized design delivers remarkable results in the following aspects: under sustained casting conditions characterized by high casting speeds and high pressure, the mechanism provides continuous, stable, and sufficient clamping force. This effectively controls the seam gap between the mold copper plates, preventing gap opening due to clamping force fluctuations and thereby fundamentally eliminating the risk of sticker breakout caused by molten steel penetration. The stable mechanical constraint also ensures the accuracy and consistency of the mold cavity geometry, creating favorable conditions for the uniform growth of the initial solidifying shell of the slab. This is directly reflected in a significant improvement in the regularity and uniformity of oscillation marks on the slab surface, laying a crucial foundation for subsequent production of high-surface-quality products. Furthermore, the hydraulic stroke is optimally set at 5–8 mm. This design provides adequate maintenance opening while strictly limiting the additional compression of the disk springs to their optimal working range (<0.35 *h*_0_). Long-term operational monitoring indicates no significant performance degradation or failure of the disk spring set. Consequently, the maintenance cycle of the entire mechanism has been extended, and its operational reliability has received high recognition from the end-user.

### 3.2. Industrial Application Performance of the Outer-Chamfered Narrow-Face Copper Plate

**Figure 8:**
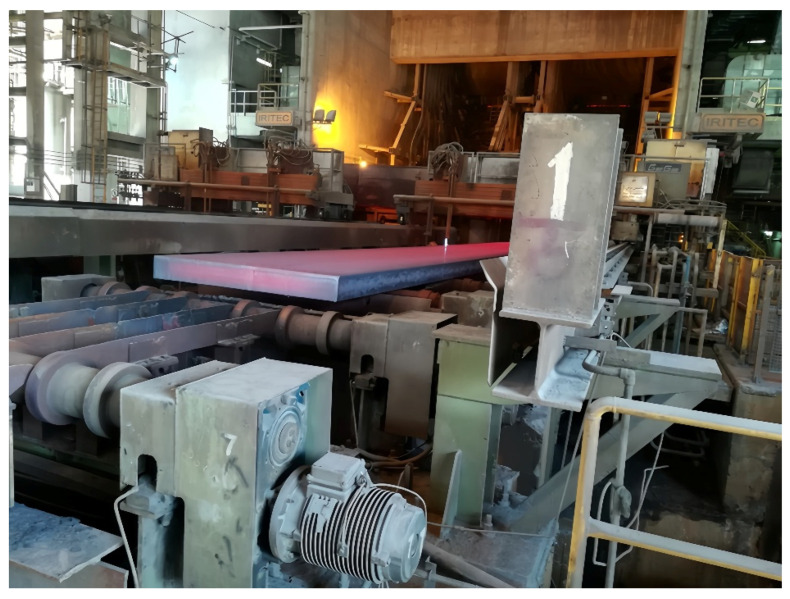
The retrofitted conventional slab production line.

To verify the industrial applicability of the newly designed outer-chamfered narrow-face copper plate, this study implemented it on a two-strand slab continuous caster at a steel plant in Europe. This caster primarily produces crack-sensitive steel grades, including peritectic steel 3034 (corresponding to the domestic grade Q215 in China), as shown in Figure 8. Since the completion of the technological upgrade and its commissioning in 2012, long-term production practice for slabs with a cross-section of 200 × 1630 mm has fully demonstrated the comprehensive superiority of this design.

**Figure 9:**
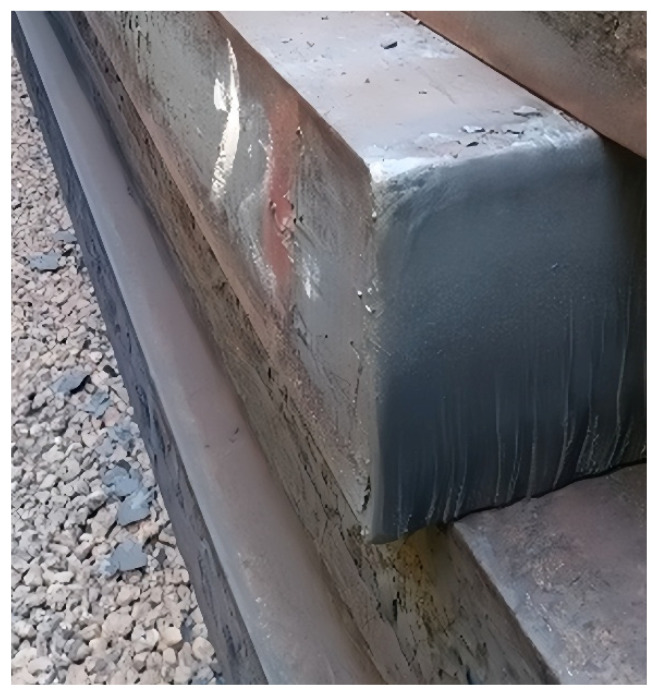
Physical photo of high-quality slab without defect.

The results indicate that after adopting the outer-chamfered copper plates, the surface quality of the slab narrow faces significantly improved, with the corner areas becoming smooth and flat (as shown in Figure 9). During continuous production, defects such as longitudinal corner cracks, transverse corner cracks, star cracks, or depressions no longer occurred, thereby completely resolving the long-standing issue of corner cracks that had constrained the production of peritectic steels on this line. Measurement data further confirmed that the slabs produced using this design are entirely consistent in dimensional specifications (width, thickness, and contour) with those produced using traditional right-angle copper plates, demonstrating high dimensional regularity. Consequently, seamless integration with the existing downstream process can be achieved without adjusting subsequent rolling parameters. Furthermore, compared to previously attempted internal chamfer solutions, the outer-chamfer design effectively mitigates abnormal wear at the lower corners of the copper plates by fully preserving the structural integrity of the working face. Actual production statistics show that the service life of the narrow-face copper plates has been significantly extended, and replacement frequency has been reduced, thereby directly lowering the production cost per ton of steel and earning high recognition from the end-user.

For crack-sensitive steel grades, the externally chamfered copper plate effectively reduces or eliminates slab corner defects while also mitigating wear on the copper plate. The service life of the narrow-face copper plate is extended from 70,000 tons to 85,000 tons, representing an increase of nearly 20%. In addition, the reduction in corner defects improves metallic yield by approximately 0.1%.

### 3.3. Discussion of the Systemic Improvement Mechanism for Slab Quality Through Coordinated Optimization

The effectiveness achieved in this study does not stem from simple improvements to isolated components but is the inherent outcome of a systematic and coordinated optimization of the two core functions of the mold: “mechanical constraint” and “heat flow control.” The optimized clamping mechanism provides a solid and stable mechanical framework for the entire system, ensuring the tight fit and dimensional accuracy of the wide- and narrow-face copper plates under demanding operating conditions. This establishes an indispensable prerequisite for any refined design aimed at improving heat transfer, such as the outer chamfer. Insufficient clamping force leading to seam variation would directly interfere with the preset cooling path and heat conduction process, rendering heat transfer optimization ineffective. Simultaneously, the outer-chamfered narrow-face copper plate directly addresses the thermodynamic root cause of slab defects. By constructing a controllable gradient thermal resistance at the corner, it transforms the original two-dimensional “intense cooling” into a gentle “gradual cooling,” thereby suppressing crack initiation caused by thermal stress concentration at the source. More critically, these two elements constitute a mutually reinforcing closed-loop system: the stable mechanical structure ensures the stable formation and effective function of the thermal resistance field designed by the outer chamfer. In turn, the resulting more uniform initial solidifying shell reduces the asymmetric load on the copper plates caused by uneven solidification shrinkage, further consolidating mechanical stability. This positive interaction and synergy between the mechanical and thermal dimensions fundamentally resolve the inherent conflict in traditional designs between “maintaining structural strength” and “optimizing heat transfer uniformity.”

The industrial application results fully confirm that the integrated mold optimization design scheme proposed in this paper, through the coordinated innovation of the clamping mechanism and the outer-chamfered copper plate, achieves breakthroughs in both mechanical reliability and solidification thermodynamics. This solution not only provides an efficient, reliable, and cost-effective equipment solution for the high-quality continuous casting of highly crack-sensitive steel grades but also offers a new perspective of “systematic coordinated design” for the future development of continuous-casting mold technology.

## 4. Conclusions

(1) This study proposes an integrated optimization scheme for key mold components. The designed disk spring–hydraulic clamping mechanism provides robust and reliable clamping force for wide-slab casting, ensuring structural integrity.
(2) Based on solidification principles, an outer chamfer design for the narrow-face copper plate is proposed and validated. By introducing a controlled and linearly increasing thermal resistance, this design promotes uniform and progressive cooling of the slab corner.
(3) Practical application demonstrates that for crack-sensitive steel grades, the externally chamfered copper plate effectively reduces corner defects and copper plate wear, extends narrow-face service life by nearly 20%, and improves metallic yield by approximately 0.1%.
(4) The optimization methodology and component design scheme detailed in this study provide an effective technical approach for improving slab surface quality and the economic performance of the continuous caster, offering significant value for engineering practice and future mold design.

## Figures and Tables

**Table 1:** Structural parameters of the mold wide-face copper plate.

*H* (mm)	*H*_0_ (mm)	*a* (mm)	*b* (mm)	*c* (mm)	*f* (mm)	*g* (mm)	*j* (mm)	*k* (mm)
1050	700	880	615	265	229	386	435	180

**Table 2:** Calculated stress parameters.

*F_V_*_0_ (kgf)	*P_A_*_0_ (kgf)	*P_B_*_0_ (kgf)	*N_S_* (kgf)	*P_AN_* (kgf)	*P_BN_* (kgf)	*F_S_*_1_ (N)	*F_S_*_2_ (N)
7717.5	1129.4	2729.4	960	178.7	301.3	19,621.5	45,460.5

**Table 3:** A100-1-type single disk spring performance parameter list.

O.D, *D* (mm)	I.D, *d* (mm)	Thickness, *t* (mm)	Max Deflection, *h*_0_ (mm)	Free Height, *H*_0_ (mm)	Deflection *f* ≈ 0.75 *h*_0_	
					Deflection, *f* (mm)	Load, *F* (N)
100	51	6	2.2	8.2	1.65	48,000

## Data Availability

The original contributions presented in this study are included in the article. Further inquiries can be directed to the corresponding author.
